# Mitochondrial and nuclear phylogenetic analysis with Sanger and next-generation sequencing shows that, in Área de Conservación Guanacaste, northwestern Costa Rica, the skipper butterfly named *Urbanus belli* (family Hesperiidae) comprises three morphologically cryptic species

**DOI:** 10.1186/1471-2148-14-153

**Published:** 2014-07-09

**Authors:** Claudia Bertrand, Daniel H Janzen, Winnie Hallwachs, John M Burns, Joel F Gibson, Shadi Shokralla, Mehrdad Hajibabaei

**Affiliations:** 1 Biodiversity Institute of Ontario & Department of Integrative Biology, University of Guelph, Guelph, Ontario N1G 2W1, Canada; 2 Department of Biology, University of Pennsylvania, Philadelphia, Pennsylvania 19104, USA; 3 Department of Entomology, National Museum of Natural History, Smithsonian Institution, P.O.Box 37012, Washington, DC 20013-7012, USA; 4 Department of Microbiology, Mansoura University, 35516 Mansoura, Egypt

**Keywords:** Lepidoptera, Intragenomic variation, Non-metric multi-dimensional scaling, Phylogeny, DNA barcoding

## Abstract

**Background:**

Skipper butterflies (Hesperiidae) are a relatively well-studied family of Lepidoptera. However, a combination of DNA barcodes, morphology, and natural history data has revealed several cryptic species complexes within them. Here, we investigate three DNA barcode lineages of what has been identified as *Urbanus belli* (Hesperiidae, Eudaminae) in Área de Conservación Guanacaste (ACG), northwestern Costa Rica.

**Results:**

Although no morphological traits appear to distinguish among the three, congruent nuclear and mitochondrial lineage patterns show that “*Urbanus belli*” in ACG is a complex of three sympatric species*.* A single strain of *Wolbachia* present in two of the three cryptic species indicates that *Urbanus segnestami* Burns (formerly *Urbanus* belliDHJ01), *Urbanus bernikerni* Burns (formerly *Urbanus* belliDHJ02), and *Urbanus ehakernae* Burns (formerly *Urbanus* belliDHJ03) may be biologically separated by *Wolbachia*, as well as by their genetics. Use of parallel sequencing through 454-pyrosequencing improved the utility of *ITS2* as a phylogenetic marker and permitted examination of the intra- and interlineage relationships of *ITS2* variants within the species complex. Interlineage, intralineage and intragenomic compensatory base pair changes were discovered in the secondary structure of *ITS2*.

**Conclusion:**

These findings corroborate the existence of three cryptic species. Our confirmation of a novel cryptic species complex, initially suggested by DNA barcode lineages, argues for using a multi-marker approach coupled with next-generation sequencing for exploration of other suspected species complexes.

## Background

The identification of species is essential for robust biodiversity estimates and a wide range of biological investigations with socio-economic applications. For example, the inability to accurately detect species has historically led to costly invasive species outbreaks and sub-optimal conservation practices for species at risk [1-3]. Herein we focus on “cryptic” species and define them according to Bickford’s overarching definition: two or more distinct species that are erroneously classified under one species name [1]. Morphologically cryptic species—those species that lack anatomical differences—require additional lines of evidence for species identification beyond traditional measures, including but not limited to: ecological distinctiveness, behavioral differences, morphological dissimilarity in undescribed life stages, and molecular divergences e.g., [4-6]. This study focuses on the latter and, more specifically, utilizes DNA barcode divergences as initial proxies for distinguishing cryptic species. DNA barcoding utilizes a short, standardized gene region to identify species; this study uses the 658 bp region of the cytochrome *c* oxidase I gene (*COI*), chosen as a DNA barcode for animal life [7]. Since 2003, DNA barcoding has been applied to a long-standing morphological and ecological inventory of Lepidoptera from the 140,000 ha terrestrial portion of Área de Conservación Guanacaste (ACG), northwestern Costa Rica (http://www.acguanacaste.ac.cr).

The ACG Lepidoptera inventory is comprehensively collecting and cataloging ancillary data including: DNA barcodes, food plants, parasitoids, fine-scale geographic and ecosystem data, and adult and caterpillar morphology [8] (http://janzen.sas.upenn.edu). Numerous instances of otherwise unrecognized species diversity have been discovered within the ACG. Several studies have focused on cryptic species complexes within the skipper butterflies (Hesperiidae) [9-13].

The discovery and characterization of species of hesperiid butterflies in ACG includes one of the most highly cited studies using DNA barcoding [13]. This study involved the *Astraptes fulgerator* (Walch, 1775) species complex and found ten distinct DNA barcode lineages. Although, some of these lineages displayed low sequence divergence (e.g., less than 1%), they were confirmed as species through the strong correlation of barcodes with distinct food plants, caterpillar coloration, some subtle differences in adult coloration and size, and ecological preferences [13].

In cases where cryptic species have been detected in Lepidoptera, natural history traits that correlate with *COI* lineages are key diagnostic characters. In this study, we explore a novel case where natural history traits appear to be absent, yet marked (i.e., more than 2%) DNA barcode divergences are present. We focus our efforts on what has been called “*Urbanus belli”*[11] in the ACG and explore the utility of a genetic framework to identify morphologically cryptic species.

**Figure 1:**
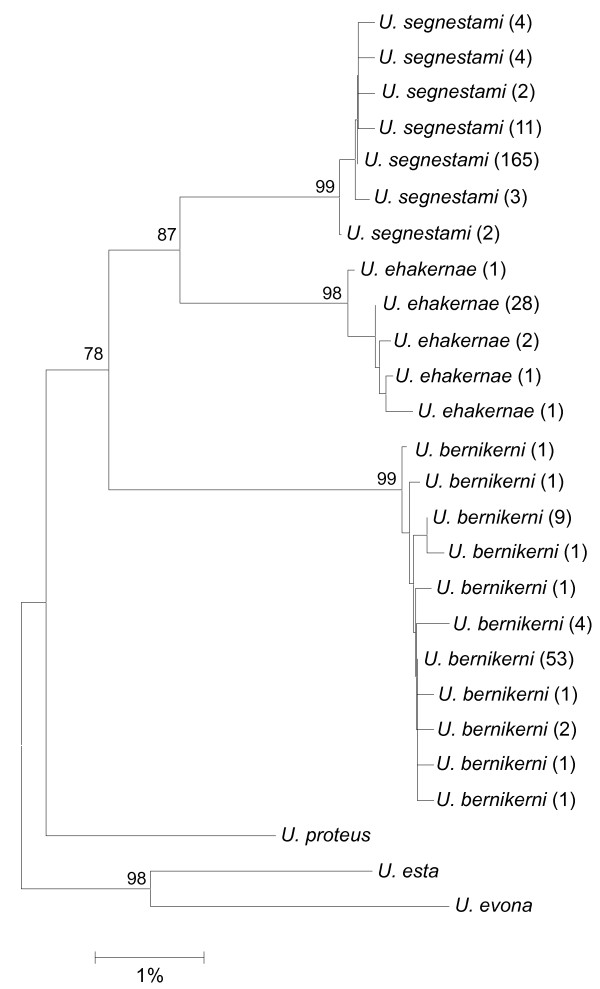
**NJ-tree of 299 *Urbanus bernikerni *complex sequences from BOLD (**http://boldsystems.org**).** Sequence divergence was estimated using the Kimura two-parameter method. Nodal support is based on 1000 bootstrap replicates. Scale bar represents number of substitutions per site. Numbers in parentheses are the number of individuals with each kind of haplotype.

**Figure 2:**
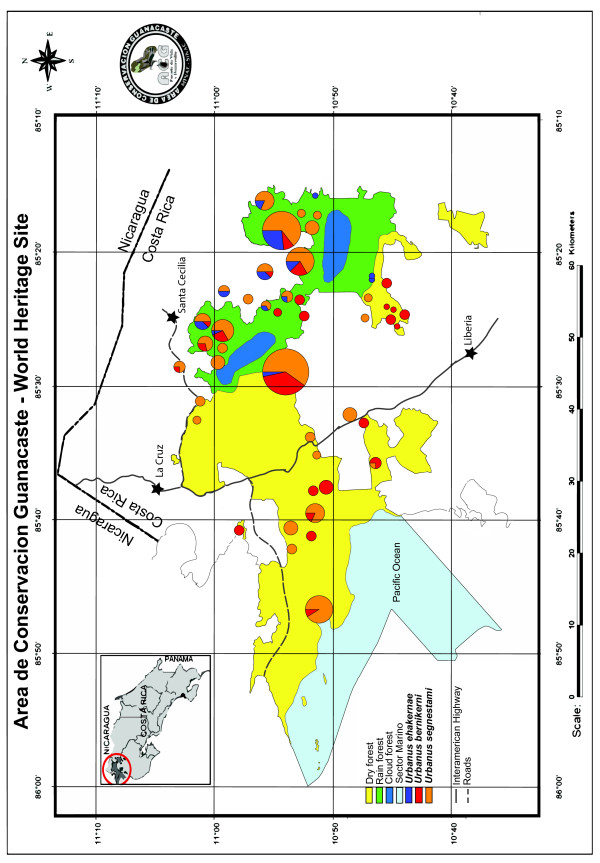
**Map of the ACG illustrating the distribution of the three species in the *Urbanus bernikerni *complex.***Urbanus segnestami* (orange circles) has the most prominent presence in dry forest (Pacific side of Cordillera Guanacaste), but extends throughout the rain forest (mostly on the Atlantic side of Cordillera Guanacaste) part of ACG as well. *Urbanus bernikerni* (red) has its most prominent presence in the rain forest, but extends well into dry forest. *Urbanus ehakernae* (blue) is exclusively rain forest; the collections at Pailas, which appear to be in the Guanacaste dry forest part of ACG, are actually in a tongue of northeastern rain forest wrapping around the Rincon de la Vieja volcanic complex.

“*Urbanus belli*” is viewed as well-categorized taxonomically and ecologically as a single species that has become specialized to feed on the leaves of tropical herbaceous or shrubby Asteraceae, i.e., composites [11] (http://janzen.sas.upenn.edu). Within the small area of ACG, this skipper butterfly contains three distinct DNA barcode lineages that have temporarily been called *Urbanus* belliDHJ01, *Urbanus* belliDHJ02, and *Urbanus* belliDHJ03 in [11]. They are separated from each other by ~3-5% *COI* sequence divergence (Figure 1). As mentioned previously, these three lineages lack apparent diagnostic morphological and natural history characters, other than that one of them, within ACG, is restricted to rain forest, where all three lineages occur naturally (Figure 2). Food plant lists overlap among all three lineages (Table 1).

**Table 1:** **325 adults of the** ***Urbanus bernikerni *** **complex reared from wild-caught caterpillars in Área de Conservación Guanacaste, caterpillar food plants and ecosystems, as of 31 Dec 2011**

**Food plant**	***Urbanus signestami***	***Urbanus bernikerni***	***Urbanus ehakernae***
**Asteraceae**			
Asteraceae *1454*	3	1	0
* Baltimora recta*	2	0	0
* Calea urticifolia*	10	2	0
* Clibadium leiocarpum*	3	1	2
* Clibadium pittieri*	18	4	8
* Clibadium surinamensis*	2	1	2
* Lasianthaea fruticosa*	1	0	0
* Lepidaploa tortuosa*	1	1	0
* Melanthera nivea*	75	43	3
* Otopappus verbesinoides*	3	0	0
* Salmea scandens*	5	1	0
* Verbesina ovatifolia*	6	8	0
* Viguiera dentata*	1	1	0
* Zexmenia virgulta*	75	23	19
**Ecosystem**	dry forest and rain forest	dry forest and rain forest	rain forest

To test the hypothesis that “*Urbanus belli”* in the ACG is a complex of three cryptic species, as suggested by *COI* data (Figure 1), we compared lineage patterns across three additional molecular markers including: another mitochondrial gene (*cytochrome b*; *cytb*), a protein-coding nuclear gene (*elongation factor subunit I alpha*; *EF1*α), and a nuclear ribosomal region (*Internal transcribed spacer region II*; *ITS2*), through the construction of phylogenetic trees. Given the high copy number and varying levels of intragenomic heterogeneity of the *ITS2*[14,15], Next-Generation Sequencing (NGS) was used to obtain sequences of *ITS2* from each specimen and non-metric multidimensional scaling (nMDS) was used to analyze intragenomic, intraspecific, and interspecific sequence clustering.

Additional analyses, including compensatory base changes (CBCs) in *ITS2* secondary structure and a *Wolbachia* endosymbiosis assay, were performed to supplement results in light of the absence of natural history characters. CBCs in the secondary structure of *ITS2* have been found to correlate strongly with distinct biological species of plants and fungi and as such, have been proposed as a molecular indicator of biological species [15-17]. The occurrence of a single CBC has been shown to correlate with distinct species of plants 93.11% of the time [16,17]. Lastly, the presence of *Wolbachia* endosymbionts was tested by using the *Wolbachia surface protein (wsp)* marker followed by Multi Locus Sequence Typing (MLST) [18]. *Wolbachia* are known to impact insect evolution by altering host reproduction. The most commonly reported mechanism is cytoplasmic incompatibility [19], the inability of infected males to reproduce successfully with uninfected females. Cytoplasmic incompatibility has been a factor in the reduction of gene flow between infected and non-infected populations, populations infected with different strains of *Wolbachia,* and populations infected with the same strain of *Wolbachia*[20]. By including this analysis, we begin to explore the role that *Wolbachia* infections can play as potential reinforcing mechanisms for species’ isolation [20].

## Methods

### Specimens

Wild caterpillars were collected from ACG, reared to adults, and added to the ongoing, comprehensive inventory of Lepidoptera, with ancillary data, including locality, food plant species, sex, and parasitoids [8,21]. Eight hundred and fifty wild-caught “*Urbanus belli*” caterpillars have been reared by the ACG inventory, and 325 of the resulting adults have now been barcoded. Table 1 provides ancillary data for all 325 adults. Fifty-one DNA barcoded individuals were selected from the Barcode of Life Data Systems (BOLD) [22] for investigation in this study. All specimens have been morphologically identified as “*Urbanus belli*.”

### Molecular analysis

DNA was extracted from a single leg of 51 specimens, using a Nucleospin Tissue Kit (Macherey-Nagel Inc., Bethlehem, PA, USA). PCR amplification and sequencing was performed for four gene regions: *COI, cytb, EF1*α, and *ITS2* (Table 2, amplification primers). *EF1*α was amplified using four primer sets that overlapped by 20-30 bp (Table 2). *COI*, *cytb*, and each fragment of *EF1*α were amplified in 25 μL reactions containing final concentrations of 0.2 mM of dNTPs, 0.2 μM of each primer, 0.024 U/μL *Taq* DNA polymerase (Invitrogen, Life Technologies, Burlington, ON, Canada), 20 μM of Tris–HCl, 50 μM KCl, 2.5 mM MgCl_2_, and 30–100 ng of DNA template. The PCR thermal cycling conditions varied by marker with *COI* and *EF1*α: 94°C for 2 min., 40 cycles (94°C for 30 sec., 51°C for 40 sec., 72°C for 1 min.), 72°C for 5 min. Conditions for *Cytb* were: 94°C for 2 min., 35 cycles (94°C for 30 sec., 48°C for 1 min., 72°C for 40 sec.), 72°C for 1 min (primer information see Table 2).

**Table 2:** Description of PCR primers used in this study

**Target**	**F primer**	**Primer sequence (5′-3′)**	**R primer**	**Primer sequence (5′-3′)**	**bp**	**Ref.**
*COI*	LepF	ATTCAACCAATCATAAAGATATTGG	LepR	TAAACTTCTGGATGTCCAAAAAATCA	658	[12]
cyt*b*	40 F	TGAATCTGAGGAGGGTTTGCIGT	560R	TCTACTGGGCGGGCTCCAATTCA	499	
*EF1*α	Starsky	CACATYATTGTCGTSATYGG	Hutch	CTTGATGAAATCYCTGTGTCC	238	[23]
*EF1*α	Bo	GCTGAGCGYGARCGTGGTATCAC	Luke	CATRTTGTCKCCGTGCCAKCC	367	[23]
*EF1*α	Laverne	GAGGAAATYAARAAGGAAG	Verdi	GACACCAGTTTCAACTCTGCC	267	[24]
*EF1*α	BJ	CARGACGTATACAAAATCGG	Bear	GCAATGTGRGCIGTGTGGCA	319	[24]
*ITS2*	*ITS2*_d1F	GAACTGCAGGACACATGAAC	ITS4	TCCTCCGCTTATTGATATGC	variable	[25]

*ITS2* was amplified in 25 μL reactions containing final concentrations of 0.2 mM of dNTPs, 0.4 μM of each primer, 0.024 U/μL*Taq* DNA polymerase, 20 μM of Tris–HCl, 50 μM KCl, 1.5 mM MgCl_2_, and 30-100 ng of DNA template. The PCR thermal cycling conditions were 94°C for 5 min., 40 cycles (94°C for 1 min., 53°C for 1 min., 72°C for 1 min.), 72°C for 10 min.

The PCR results were visualized on a 1.5% agarose gel stained with ethidium bromide. Any PCR products that showed a band on the agarose gel underwent cycle sequencing reactions with the following protocol: 0.25 μL of Dye terminator mix v3.1, 1.875 μl of 5× Sequencing Buffer, 1 μL of 10 μM Primer, 5.875 μL of H_2_0, and 2.0 μL of PCR product for a final volume of 11 μL. The cycle sequencing thermocycling protocol was as follows: 96°C for 2 min., 30 cycles (96°C for 30 sec., 55°C for 15 sec., 60°C for 4 min.). Cycle sequenced products were resolved on an ABI 3730XL sequencer (Applied Biosystems, Foster City, CA, USA).

*EF1*α sequences were heterogeneous, showing double peaks in sequence chromatograms. Consequently, polymorphic (heterogeneous) bases were scored using the IUPAC code for ambiguous bases. Sequences were edited using CodonCode Sequence V3.1.5 (CodonCode, Dedham, MA, USA) software, aligned using CLUSTAL W [26], and further manual inspection was done in MEGA 5.0 [27].

Sequences from *Urbanus proteus* (Linnaeus, 1758)*, Urbanus esmeraldus* (Butler, 1877), *Urbanus evona* Evans, 1952, and *Urbanus esta* Evans, 1952 were generated using the same protocols and were included as outgroups.

MrModelTest2.3 [28] was used to find the best nucleotide substitution model prior to Bayesian analysis. The GTR + G model was selected for both *COI* and *cytb*, while K80 + G was selected for *EF1*α. MrBayes v3.1.2 [29] was used to generate Bayesian gene trees for each marker. A total of 56 sequences were included in analysis of *COI*, 54 for *cytb*, and 30 for *EF1*α.

Sequencing success of the four amplified regions of *EF1a* was variable (Table 3). To maximize the number of specimens included in the Bayesian analysis, a sequence length of 541 bp was utilized. For quality assurance, sequence that had greater than 1% basepair ambiguities were excluded.

**Table 3:** **Sampling of *Urbanus bernikerni *** **complex specimens and gene region success**

**Voucher code**	**Identification**	***COI *** **seq. length**	**n**	***cytb *** **seq. length**	**n**	***EF1a *** **seq. length**	**n/polymorphism**	***ITS2 *** **seq. length**
08-SRNP-4727	*Urbanus segnestami*	658	0n	499	0n	0	N/A	>200 bp
06-SRNP-47955	*Urbanus segnestami*	658	0n	499	0n	316	0n	>200 bp
06-SRNP-46570	*Urbanus segnestami*	657	0n	499	0n	1030	5n	>200 bp
06-SRNP-47304	*Urbanus segnestami*	632	0n	499	0n	316	2n	>200 bp
07-SRNP-41187	*Urbanus segnestami*	642	1n	499	0n	316	2n	>200 bp
08-SRNP-23800	*Urbanus segnestami*	658	0n	475	0n	315	2n	>200 bp
08-SRNP-5848	*Urbanus segnestami*	658	0n	456	0n	316	5n	>200 bp
08-SRNP-65935	*Urbanus segnestami*	658	0n	443	0n	1045	2n	>200 bp
08-SRNP-1439	*Urbanus segnestami*	658	0n	499	0n	1030	1n	>200 bp
08-SRNP-65153	*Urbanus segnestami*	658	0n	441	0n	1045	1n	0
07-SRNP-65582	*Urbanus segnestami*	658	0n	475	0n	1045	8n	>200 bp
07-SRNP-33232	*Urbanus segnestami*	658	0n	429	0n	1045	5n	>200 bp
07-SRNP-1119	*Urbanus segnestami*	658	0n	476	0n	1045	2n	0
07-SRNP-42421	*Urbanus segnestami*	658	0n	475	0n	1045	2n	0
07-SRNP-45090	*Urbanus segnestami*	658	0n	499	0n	933	7n	0
08-SRNP-24278	*Urbanus segnestami*	658	0n	0	N/A	0	N/A	>200 bp
06-SRNP-47801	*Urbanus bernikerni*	597	0n	499	0n	1030	0n	>200 bp
07-SRNP-30615	*Urbanus bernikerni*	658	0n	465	0n	541	3n	>200 bp
07-SRNP-20318	*Urbanus bernikerni*	658	0n	444	0n	0	N/A	>200 bp
06-SRNP-18276	*Urbanus bernikerni*	658	0n	499	0n	0	N/A	>200 bp
07-SRNP-57868	*Urbanus bernikerni*	658	0n	445	0n	541	0n	>200 bp
03-SRNP-12634.1	*Urbanus bernikerni*	658	0n	499	0n	0	N/A	0
06-SRNP-47816	*Urbanus bernikerni*	658	0n	499	0n	1030	2n	>200 bp
07-SRNP-45071	*Urbanus bernikerni*	655	0n	499	0n	316	0n	>200 bp
01-SRNP-4475	*Urbanus bernikerni*	658	0n	499	0n	316	1n	>200 bp
06-SRNP-3621	*Urbanus bernikerni*	658	0n	499	0n	0	N/A	0
07-SRNP-40856	*Urbanus bernikerni*	658	0n	499	0n	930	0n	0
07-SRNP-56547	*Urbanus bernikerni*	658	0n	443	0n	260	3n	>200 bp
07-SRNP-56829	*Urbanus bernikerni*	658	0n	446	0n	0	N/A	>200 bp
07-SRNP-30613	*Urbanus bernikerni*	658	0n	499	0n	541	3n	>200 bp
07-SRNP-40676	*Urbanus bernikerni*	658	0n	474	0n	541	1n	0
06-SRNP-47817	*Urbanus bernikerni*	658	0n	499	0n	0	N/A	0
06-SRNP-46460	*Urbanus bernikerni*	657	0n	499	0n	316	0n	>200 bp
06-SRNP-46639	*Urbanus bernikerni*	658	0n	499	0n	541	1n	0
06-SRNP-6511	*Urbanus bernikerni*	657	0n	499	0n	316	1n	0
06-SRNP-46572	*Urbanus bernikerni*	658	0n	499	0n	1030	3n	>200 bp
05-SRNP-1832	*Urbanus bernikerni*	658	0n	499	0n	0	N/A	0
02-SRNP-954	*Urbanus bernikerni*	658	0n	499	0n	316	1n	0
06-SRNP-65065	*Urbanus ehakernae*	649	0n	499	0n	1045	4n	0
07-SRNP-33182	*Urbanus ehakernae*	648	0n	499	0n	1045	3n	>200 bp
08-SRNP-65993	*Urbanus ehakernae*	658	0n	499	0n	1045	1n	>200 bp
08-SRNP-66028	*Urbanus ehakernae*	658	0n	499	0n	1045	4n	>200 bp
07-SRNP-33183	*Urbanus ehakernae*	658	0n	499	0n	541	0n	>200 bp
07-SRNP-33184	*Urbanus ehakernae*	658	0n	499	0n	1045	5n	>200 bp
07-SRNP-287	*Urbanus ehakernae*	658	0n	499	0n	316	0n	0
07-SRNP-40761	*Urbanus ehakernae*	655	0n	499	0n	1045	1n	>200 bp
06-SRNP-46887	*Urbanus ehakernae*	657	0n	499	0n	316	5n	>200 bp
06-SRNP-46891	*Urbanus ehakernae*	657	0n	499	0n	1030	11n	>200 bp
05-SRNP-40545	*Urbanus ehakernae*	658	0n	499	0n	541	0n	>200 bp
05-SRNP-41753	*Urbanus ehakernae*	658	0n	499	0n	316	3n	>200 bp
06-SRNP-43129	*Urbanus ehakernae*	381	0n	499	0n	1030	2n	>200 bp
07-SRNP-57988	*Urbanus evona*	658	0n	499	0n	1030	2n	0
05-SRNP-66411	*Urbanus evona*	658	0n	499	0n	851	0n	>200 bp
04-SRNP-15286	*Urbanus evona*	658	0n	498	1n	848	3n	0
07-SRNP-1369	*Urbanus proteus*	658	0n	499	0n	314	2n	0
08-SRNP-71995	*Urbanus proteus*	658	0n	499	0n	415	4n	0
08-SRNP-21596	*Urbanus esmeraldus*	658	0n	499	0n	313	3n	0
07-SRNP-57862	*Urbanus esmeraldus*	658	0n	499	0n	315	1n	0
07-SRNP-57861	*Urbanus esmeraldus*	658	0n	0	N/A	416	3n	0
06-SRNP-1984	*Urbanus esta*	658	0n	443	0n	845	6n	0
07-SRNP-56430	*Urbanus esta*	658	0n	499	0n	848	3n	0
07-SRNP-57988	*Urbanus evona*	658	0n	499	0n	1030	2n	0

Sequence length and number of ambiguous bases and/or polymorphic positions [n] are included for each specimen.

Four Monte Carlo Markov chains and a temperature of 0.2 were used for each analysis. Trees were sampled every 100 generations for 1,000,000 generations and Bayesian posterior probabilities were estimated for each node. The first 1250 trees (25%) were discarded as burn-in.

### 454-pyrosequencing analysis of *ITS2*

Thirty-nine individuals were selected for 454-pyrosequencing analysis. Multiple Identifier (MID) Tags were used to combine *ITS2* fragments from all individuals into a single 454 lane in a 16-lane sequencing run. *ITS2* fragments were PCR amplified with the MID tagged-primers using the same reaction mix mentioned above and *ITS2* was amplified under the following thermal cycling conditions: 94°C for 5 min., 40 cycles (94°C for 1 min., 53°C for 1 min., 72°C for 1 min.), 72°C for 10 min. All amplicons were sequenced on a 454 Genome Sequencer FLX System (Roche Diagnostics GmbH) following amplicon sequencing protocols (http://www.454.com). Amplicons of each sample were bi-directionally sequenced in 1/16th of a full sequencing run (70×75 picotiter plate).

Data was filtered using a 10-5-15 sliding window-phred score approach [30,31]. Sequences that passed this initial quality filtering underwent further filtering using the PRINSEQ webserver [32] to remove sequences with base ambiguities. A sequence length threshold of 200 bp was selected to preserve nucleotide variation and the number of sequences included in the analysis. Chimera detection was performed in UCHIME using the *de novo* option [33]. Forward and reverse direction sequences were reduced to variants by collapsing sequences that were 100% identical to each other, using the CD-HIT webserver [34]. As the final quality control, to further reduce potential impact of 454-pyrosequencing error, we only included variants that had 2 or more collapsed sequences. Only forward direction *ITS2* sequences were used in ordination analyses (see below) because reverse variants, after filtering, were minimal (1 to 3) for each individual. Forward direction variants of each individual were aligned in MEGA 5.0 using ClustalW with an alignment gap opening value of 7 and a gap extension value of 3, followed by manual editing.

Ordination by non-metric multidimensional scaling (nMDS), using the Bray-Curtis similarity measure [35] was performed in PRIMER v6 [36] to create a graphical representation of the intragenomic, intraspecific, and interspecific clustering of *ITS2.* A similarity matrix of variants found within each individual was created by treating every position in the sequence alignment as a separate character, and nucleotides were coded arbitrarily as A = 0, C = 1, G = 2, T = 3 [7] and gaps were treated as a fifth character, gap =4, in the matrix.

We assembled forward and reverse direction variants of *ITS2* that had at least 95% match in a 20 bp overlap. These full-length *ITS2* sequences were aligned in MEGA 5.0 and maximum parsimony (MP) trees were generated in PAUP 4b1.0 [37] with and without gaps as a fifth base. A heuristic search was performed with tree bisection reconnection (TBR) branch swapping and 1,000 random taxon addition replicates, saving no more than 100 equally parsimonious trees per replicate. One thousand bootstrap replicates were performed and 50% majority rule trees were created. Nodes were collapsed below the 50% confidence interval and only bootstrap values above 50% confidence interval were included.

### *ITS2* secondary structure and CBC analysis

Full-length *ITS2* intragenomic variants were collapsed by 100% identity to reduce redundancy of the dataset using the CD-HIT web-server [34]. Compensatory base changes (CBCs) occur when both nucleotides of a paired site mutate while the pairing itself is maintained (G-C to A-U) [38]. Secondary structure was determined for unique *ITS2* variants using the Vienna RNAfold Webserver (http://rna.tbi.univie.ac.at/cgi-bin/RNAfold.cgi). A default setting of a folding temperature at 37°C, allowing for dangling energies on both sides of a helix, using minimum free energy and partition function fold algorithms was employed. The program 4SALE [39] was used to align secondary structure and estimate CBCs between variants.

### *Wolbachia* assays

The presence of *Wolbachia* infection was established by a PCR test for the *Wolbachia* surface protein (*wsp*) in leg tissue. PCR reaction mixture and amplification protocols for the *wsp* marker are given by Baldo et al. [18]. If *wsp* PCR bands were present in some or all individuals of a DNA barcode lineage, all specimens from that lineage underwent the more extensive Multilocus Sequence Typing (MLST) assay [18] to identify the specific strain of *Wolbachia*. PCR band checks, Sanger sequencing protocols, and editing are the same as described for *COI* and *cytb.*

## Results

### Description of cryptic species

Permanent names are needed for further discussion of the three cryptic species in this study. As noted above, all three species are passing under the one name *Urbanus belli*, a species currently treated as pan-neotropical although it was described only from northern Argentina [40]. Given three cryptic species in northwestern Costa Rica, the different geologic history of Meso- and South America, and the degree to which we are finding that supposedly single species occurring in both continents actually comprise two or more related species that are much more limited in distribution (often replacing one another geographically), we believe that extending the application of the name *Urbanus belli* to ACG lowland Asteraceae-eating *Urbanus* is unwarranted.

Evans [41] correctly judged that true *Urbanus belli* is closely related to *Urbanus viterboana*[42], which in ACG is the high elevation Asteraceae-eating *Urbanus*. He went so far as to treat *Urbanus belli* as a subspecies of it with a limited, far southern distribution, recording *Urbanus viterboana belli* only from Argentina and Bolivia. However, he also described a new subspecies, *Urbanus viterboana alva*, which he recorded as pan-neotropical (Mexico to Argentina). Were *alva* an applicable epithet, it would pertain to a full species because it appears to be broadly sympatric with *Urbanus viterboana viterboana*, which Evans likewise recorded as pan-neotropical (Mexico to Bolivia). Morphology of the intricate genitalia of skippers is often diagnostic of species. Evans [41: 85–87; plate 18, figure C.13.2] described *Urbanus viterboana alva* from both the “very variable” superficial appearance of the adult and the male genitalia (which are somewhat variable but, at the same time, morphologically simple as skipper genitalia are concerned). Steinhauser [43] pointed out that the Evans dissection of the holotype of *Urbanus viterboana alva* is partial and in poor condition, and that the broken genitalic parts (comprising only the claspers) on the associated card cannot possibly have come from the holotype, so that one cannot say to what taxon the name should apply. We are disregarding the epithet *alva*.

In the Neighbour-Joining trees based on DNA barcodes of ACG skippers (Figure SI in [11]), two of the three cryptic species cluster more closely with the species *Urbanus viterboana* than with the third cryptic species. We call this four-species cluster the *Urbanus viterboana* group. Natural history evidence strongly supports this grouping: so far as is known (and amply documented in the ACG *Urbanus* caterpillar inventory [21]), caterpillars of the species of *Urbanus* that feed on dicots—and caterpillars of the species in many of its related genera as well—feed almost exclusively on legumes (Fabaceae), except for the four species of the *Urbanus viterboana* group, which eat the mature leaves of herbaceous composites (Asteraceae). These large plant families are not phylogenetically close to each other, and the skippers’ evolutionary shift in larval foodplant selection underlines their tight relationship and the distinctiveness of the *Urbanus viterboana* group.

**Figure 3:**
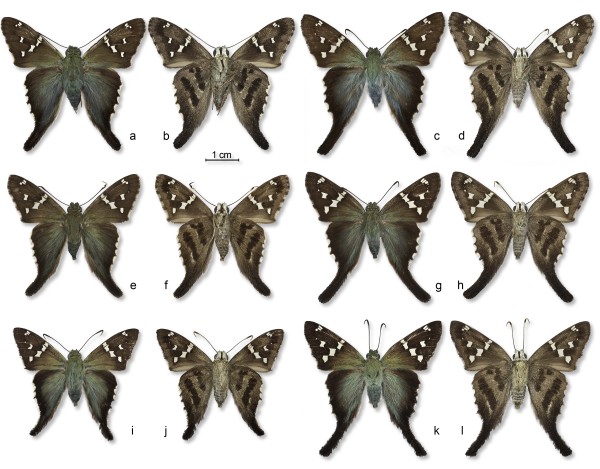
**Photographs of type specimens.** Reared males (columns one and two, left to right) and females (columns three and four, left to right) of three new cryptic species of *Urbanus* from ACG, northwestern Costa Rica, in dorsal (left) and ventral (right) view. Males are holotypes; females are paratypes (pins and pinholes artificially removed). **a**–**d**, *Urbanus segnestami* (06-SRNP-46181, 02-SRNP-1028); **e**–**h**, *Urbanus bernikerni* (93-SRNP-3294, 93-SRNP-3098); **i**–**l**, *Urbanus ehakernae* (03-SRNP-10927, 01-SRNP-22184) (voucher code of each specimen in parentheses).

The three cryptic species initially revealed by barcodes are superficially indistinguishable (Figure 3) and *Urbanus viterboana* itself is not much different (see images in [21]). Although the uppersides of their wings differ greatly from the undersides, this difference is similar across all three species (Figure 3). Hindwing tails are longer in females (Figure 3c, d, g, h, k, l) than in males (Figure 3a, b, e, f, i, j), but both sexes express the same slight variations in color pattern. The most evident variation is at the top end of the innermost of the two dark brown bands on the ventral hindwing, where the phenotype ranges from two separate spots (Figure 3d) to variable degrees of union with one another and with the band itself. This trio of cryptic species is here designated the *Urbanus bernikerni* complex of the *Urbanus viterboana* group. Because the structural color on the upperside of its wings is much more blue than green, *Urbanus viterboana* is superficially separable from the species of the *Urbanus bernikerni* complex (at least when specimens are in good condition). Likewise, the hindwing tails of *Urbanus viterboana* are slightly longer than are those of the *Urbanus bernikerni* complex. *Urbanus viterboana* lives at higher elevations (at least 900 m) than do the cryptic species.

Probing the possibility that species of the *Urbanus bernikerni* complex differ in communicative characters, such as ultraviolet reflectance patterns of the wings or pheromones released by a secondary sex character on the forewing of males, was beyond the scope of our study.

The following original taxonomic descriptions of these look-alike species are necessarily brief (Table 4). Except for the barcodes, the molecular characters and the analysis and interpretation of them are too complex to express concisely. The characters require discussion, and they have their limitations (see subsequent sections of this paper). Speciation has no regard for taxonomists. However, the DNA barcodes themselves are highly conservative within each species of the *Urbanus viterboana* group and significantly different among them (Figure 1 and appendix to [11]). The holotype chosen for each new species has the predominant barcode of that species; and the barcode, comprising 658 base pairs in each case, is given. The voucher code for a specimen leads to detailed information about the stage in which that specimen was collected, what it was eating, exactly where it was found, etc.—information that is provided in the ACG inventory database [21].

**Table 4:** **Descriptions of the three new species comprising the *Urbanus bernikerni *** **complex, based on specimens reared from wild-caught caterpillars in ACG, northwestern Costa Rica, and deposited in USNM**

**Characters**	***Urbanus segnestami *** **Burns n. sp.**	***Urbanus bernikerni *** **Burns n. sp.**	***Urbanus ehakernae *** **Burns n. sp.**
**Holotype male voucher code**	06-SRNP-46181	93-SRNP-3294	03-SRNP-10927
**Holotype DNA barcode & Genbank Accession No.**	AACTTTATATTTTATTTTTGGAATT TGAGCAGGATTAATTGGAACTT CTCTAAGATTACTTATTCGAACT GAATTAGGAACTCCAGGATCTTT AATTGGAGATGATCAAATTTATA ATACTATTGTAACAGCTCACGCA TTTATTATAATTTTCTTTATAGTT ATACCTATTATAATTGGAGGATT TGGTAATTGACTAGTACCTTTAA TAATAGGAGCCCCTGATATAGCT TTCCCCCGTATAAACAATATAAG ATTTTGATTACTACCCCCATCTTT AACTTTATTAATTTCAAGAAGAAT TGTTGAAAATGGTGCTGGTACTG GATGAACAGTTTATCCCCCCCTTT CATCTAATATTGCCCATCAAGGAG CTTCTGTTGATTTAGCAATTTTTTC TCTTCATCTTGCAGGTATTTCATC AATTCTTGGAGCTATTAATTTTAT TACAACAATTATTAATATACGAAT AATAGATTATCTTTTGATCAAAT ACCATTATTTGTATGAGCTGTAG GAATTACAGCATTATTATTATTA CTTTCTTTACCTGTTTTAGCTGG AGCTATTACTATATTATTAACTG ACCGAAACTTAAATACTTCATTT TTTGATCCTGCTGGAGGAGGAG ATCCAATTCTATATCAACATTTA TTT, GU156383	AACCTTATATTTTATTTTTGGAAT TTGAGCAGGATTAATTGGAACTT CTTTAAGATTACTTATTCGAACA GAATTAGGAACCCCAGGATCTCT AATTGGAGATGATCAAATTTATA ATACTATTGTAACAGCTCATGCA TTTATTATAATTTTTTTTATAGTT ATACCTATTATAATTGGAGGATT TGGTAATTGATTAGTCCCTTTAA TAATAGGTGCTCCTGATATAGCT TTCCCCCGTATAAACAATATAAG ATTTTGATTATTACCCCCCTCATT AACTTTATTAATTTCAAGAAGAAT TGTTGAAAATGGAGCTGGTACTG GATGAACAGTTTATCCCCCTCTT TCATCTAATATTGCTCACCAAGG AGCCTCAGTTGATTTAGCAATTT TTTCCCTTCATCTCGCTGGTATTT CATCAATTCTTGGAGCTATTAAT TTTATTACAACAATTATTAATAT ACGAATTAATAGATTAACTTTTG ATCAAATACCTTTATTTGTATGA GCCGTAGGAATTACAGCATTATT ATTATTACTTTCTTTACCTGTTT TAGCTGGAGCTATTACTATATTA TTAACAGATCGAAATTTAAATAC ATCGTTTTTTGATCCTGCTGGAG GAGGAGATCCAATTTTATACCAA CATTTATTT, JQ526818	AACTTTATATTTTATTTTTGGAA TTTGAGCAGGATTAATTGGAACT TCTTTAAGATTACTTATTCGAACT GAATTAGGAACCCCAGGATCTTT AATTGGAGATGATCAAATTTATA ATACTATTGTTACAGCTCACGCAT TTATCATAATTTTTTTTATAGTTA TACCTATTATAATTGGAGGATTT GGTAATTGATTAGTACCTTTAAT AATAGGAGCCCCTGATATAGCTT TCCCTCGTATAAACAATATAAGA TTTTGATTATTACCTCCTTCTTT AACTTTATTAATTTCAAGAAGAA TTGTTGAAAATGGTGCTGGTACT GGATGAACAGTTTATCCCCCTCT TTCATCTAATATTGCCCACCAAG GAGCTTCTGTTGATTTAGCAATT TTTTCTCTTCATCTAGCAGGTAT CTCATCAATTCTTGGAGCTATTA ATTTTATTACAACAATCATTAACA TACGAATTAATAGATTATCTTTTG ATCAAATACCATTATTTGTGTGAG CTGTAGGAATTACAGCATTATTAT TATTACTTTCTTTACCTGTTTTAGC TGGAGCTATTACTATATTATTAACC GATCGAAACTTAAATACTTCATTTT TTGACCCTGCTGGAGGAGGAGAT CCAATTCTATATCAACATTTATTT, GU161980
**Paratypes**	107 males, 84 females	39 males, 36 females	16 males, 16 females
**DNA barcode cluster in NJ tree**	See Figure 1	See Figure 1	See Figure 1
**Additional molecular characters**	See subsequent text	See subsequent text	See subsequent text
**Ecosystem**	Dry forest and rain forest	Dry forest and rain forest	Rain forest
**Presence of *Wolbachia *** **strain 108 of Supergroup B**	Yes	No	Yes
**Etymology**	Named in honor of Mats Segnestam of Sigtuna, Sweden, formerly of Svenska Naturforeningen and then the Swedish governmental agency SIDA, in recognition of his intense two decades of facilitating the germination and growth of ACG as a concept and reality of conservation through non-damaging biodiversity development.	Named in honor of Bernd Kern (RIP) of Vaesterhaninge, Sweden, in recognition of his joint high energy, dedication, and enthusiasm with Eha Kern in the 1980's creation and subsequent decades of execution of Barnensregnskog (Childrens Rainforest of Sweden, http://www.barnensregnskog.se**)** in support of land purchase for ACG and support of many other Neotropical conservation projects.	Named in honor of Eha Kern of Vaesterhaninge, Sweden, in recognition of her joint high energy**,** dedication, and enthusiasm with Bernd Kern in the 1980's creation and subsequent decades of execution of Barnensregnskog (Childrens Rainforest of Sweden, http://www.barnensregnskog.se) in support of land purchase for ACG and support of many other Neotropical conservation projects.

**Table 5:** **Results of 454-pyrosequencing *ITS2 *** **from the *Urbanus bernikerni *** **complex**

**MID**	**Sample ID**	**Species**	**No. sequences**	**No. unique variants**	**No. variants (>2 seq)**	**No. sequences in variants (>2 seq)**
22	07-SRNP-30614	*Urbanus segnestami*	133	117	7	16
24	07-SRNP-33232	*Urbanus segnestami*	147	136	6	11
26	08-SRNP-1439	*Urbanus segnestami*	207	172	15	35
27	08-SRNP-65935	*Urbanus segnestami*	66	46	8	20
28	08-SRNP-5848	*Urbanus segnestami*	174	150	11	24
29	08-SRNP-23800	*Urbanus segnestami*	142	117	9	25
30	08-SRNP-24278	*Urbanus segnestami*	183	137	18	46
52	07-SRNP-41187	*Urbanus segnestami*	120	91	13	29
53	06-SRNP-47304	*Urbanus segnestami*	80	63	8	17
54	06-SRNP-47955	*Urbanus segnestami*	170	117	23	53
55	06-SRNP-46570	*Urbanus segnestami*	143	115	13	28
56	08-SRNP-4727	*Urbanus segnestami*	43	36	3	7
31	06-SRNP-18276	*Urbanus bernikerni*	106	90	8	16
32	06-SRNP-46572	*Urbanus bernikerni*	151	120	24	71
33	07-SRNP-30615	*Urbanus bernikerni*	179	132	20	47
34	07-SRNP-30613	*Urbanus bernikerni*	233	189	17	44
37	07-SRNP-20318	*Urbanus bernikerni*	30	24	3	6
38	07-SRNP-57868	*Urbanus bernikerni*	56	50	3	6
39	07-SRNP-56547	*Urbanus bernikerni*	82	73	4	9
40	07-SRNP-56829	*Urbanus bernikerni*	322	266	23	56
57	07-SRNP-45071	*Urbanus bernikerni*	129	109	8	10
64	06-SRNP-47801	*Urbanus bernikerni*	240	166	30	74
65	06-SRNP-46460	*Urbanus bernikerni*	147	115	12	32
68	05-SRNP-1832	*Urbanus bernikerni*	4	4	0	0
69	01-SRNP-4475	*Urbanus bernikerni*	74	68	3	6
41	05-SRNP-41753	*Urbanus ehakernae*	164	125	17	39
42	05-SRNP-40545	*Urbanus ehakernae*	108	88	7	20
43	06-SRNP-65065	*Urbanus ehakernae*	11	11	0	0
45	07-SRNP-40761	*Urbanus ehakernae*	144	93	21	51
46	07-SRNP-33184	*Urbanus ehakernae*	169	118	18	51
47	07-SRNP-33183	*Urbanus ehakernae*	101	74	11	27
48	07-SRNP-33182	*Urbanus ehakernae*	117	80	17	37
49	08-SRNP-66028	*Urbanus ehakernae*	106	76	10	30
50	08-SRNP-65993	*Urbanus ehakernae*	156	126	13	30
70	06-SRNP-43129	*Urbanus ehakernae*	134	91	17	34
71	06-SRNP-46891	*Urbanus ehakernae*	190	156	11	34
72	06-SRNP-46887	*Urbanus ehakernae*	293	237	26	56
73	05-SRNP-42394	*Urbanus ehakernae*	112	87	11	25

**Table 6:** CBC matrix showing the number of compensatory base changes within and between each provisional species

	***Urbanus segnestami***	***Urbanus bernikerni***	***Urbanus ehakernae***
*Urbanus segnestami*	0		
*Urbanus bernikerni*	17	0	
*Urbanus ehakernae*	10	12	4

**Table 7:** ***Wolbachia *** **BLAST results from the *wsp *** **and *MLST *** **database for the five markers: *coxA, hpcA, gatB, fstZ*, and *fbpA***

**Marker**	**Allele**	**Percentage identity match in WSP & MLST databases**
*wsp*	115	100%
*gatB*	71	98%
*coxA*	67	100%
*ftsZ*	65	99%
*hcpA*	74	99%
*fbpA*	6	98%

The table includes the allele type that was found in the BLAST search and the percentage identity match in the databases resulting in the *Wolbachia* strain 108 of Supergroup B.

As required by the International Code of Zoological Nomenclature for electronic publication of new species, this work is registered in ZooBank as (urn:lsid:zoobank.org:pub:B033EAF0-C9C9-47 F1-B4D8-FAA829F6876B), and will be electronically archived by PubMed Central (http://www.ncbi.nlm.nih.gov/pmc) once published. The date of publication will be the date of publication of this paper by BMC Evolutionary Biology (ISSN 1471–2148).

### Phylogenetic analysis of *COI, cytb*, and *EF1*α

**Figure 4:**
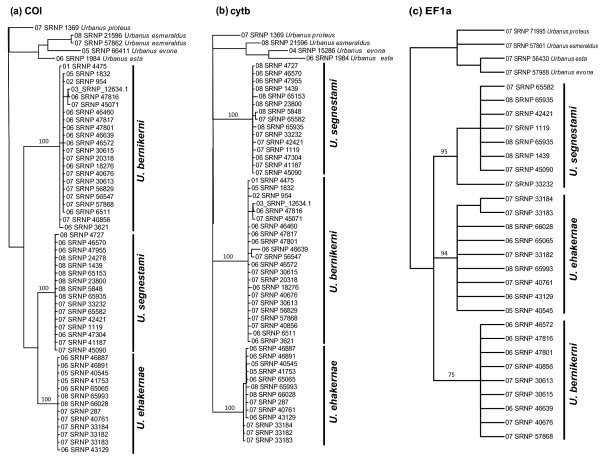
**Bayesian trees constructed from *COI *(panel a)*, cytb *(panel b)*, *and *EF1a *(panel c) sequences of the *Urbanus bernikerni *complex showing the relationship between mitochondrial and nuclear gene regions.** Posterior probabilities are presented > 70%.

Sequencing success for each marker and all specimens is shown in Table 3. Sequences were also submitted to the Dryad repository and can be found at doi:10.5061/dryad.pj561. The results of the Bayesian analyses of *COI*, *cytb*, and *EF1*α show three supported clades (Figure 4). These clades are congruent with the species boundaries suggested by DNA barcode data (Figure 1).

### Analysis of *ITS2* sequences

**Figure 5:**
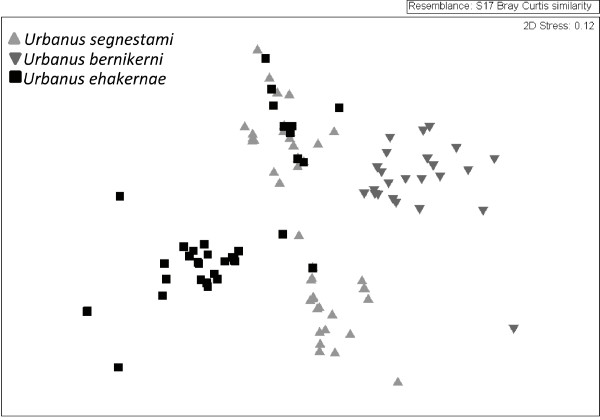
**nMDS graph displaying the intra-genomic variation of *ITS2 *** **sequences of the *Urbanus bernikerni *** **complex.**

The 39 individuals successfully 454-pyrosequenced for *ITS2* produced 5,315 sequence reads, which after filtering consisted of 303 variants (Table 5). The nMDS plot (Figure 5) shows distinct separation of *Urbanus bernikerni* from *Urbanus segnestami* and *Urbanus ehakernae.* Two clusters are observed for *Urbanus segnestami*, which highlights the level of apparently intraspecific variability that is found within this lineage, though the possibility of yet more phylogenetic structure in this population cannot be unambiguously discarded. Two individuals, 06-SRNP-46887 and 06-SRNP-46891 of *Urbanus ehakernae*, each possess a variant (06-SRNP-46887_a, 06-SRNP-46891_d) that falls into the *Urbanus segnestami* cluster. These two individuals are most likely siblings. Both were found at the very western-most edge of the distribution of *Urbanus ehakernae* as it protrudes into the more encompassing “dry forest to rain forest” distribution of *Urbanus segnestami.*

**Figure 6:**
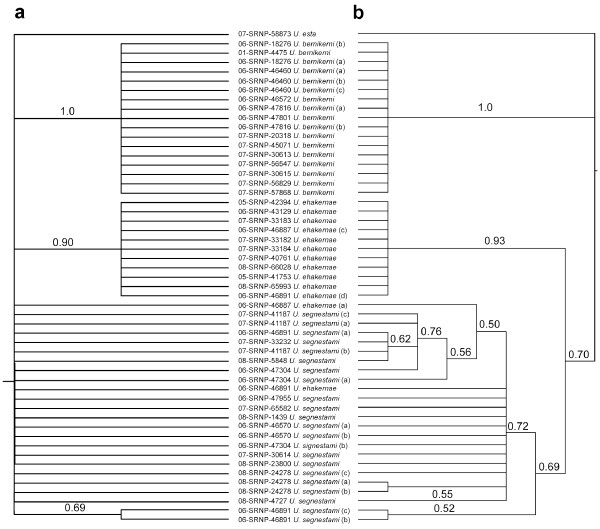
**Maximum Parsimony cladogram constructed from *ITS2 *sequences of the *Urbanus bernikerni *complex.** Bootstrap values are presented above 50%. Including gaps as a fifth character (panel **a**); dismissing gaps as characters (panel **b**).

The alignment of *ITS2* sequences in the MP analyses with gaps treated as missing data had 28 parsimony-informative characters (Figure 6a), while the *ITS2* alignment with gaps treated as a fifth character had 41 parsimony-informative characters (Figure 6b). The MP analysis of the *ITS2* alignment that included gaps as fifth characters (CI = 0.853, RI 0.937) resulted in 144 most-parsimonious trees; and the 50% majority-rule consensus tree supports reciprocal monophyly of *Urbanus bernikerni,* but shows *Urbanus ehakernae* to be paraphyletic to *Urbanus segnestami* (Figure 6b). The paraphyly is caused by the 2 variants mentioned above, 06-SRNP-46887_a and 06-SRNP-46891_d (Figure 6b). If gaps are not considered as a fifth base, the MP analysis resulted in 770 most-parsimonious trees (CI = 0.861, RI = 0.954); and the 50% majority-rule consensus tree shows less than 50% support for the *Urbanus segnestami* node (Figure 6a).

### *ITS2* secondary structure & compensatory base changes

**Figure 7:**
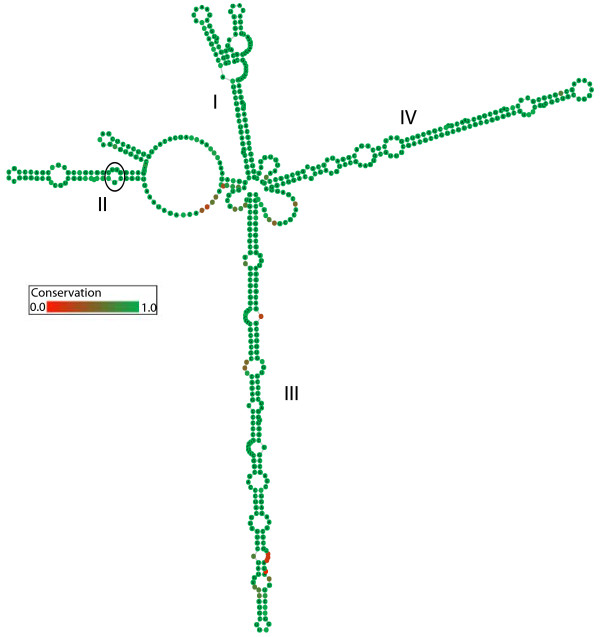
**Conserved *ITS2 *secondary structure for variants of the *Urbanus bernikerni *complex.** Helices are numbered accordingly. The U-U mismatch typically found in Eukaryotes is outlined with a black circle in helix II. The complete structure represents the 51% consensus of aligned structures with gaps. Degree of secondary structure conservation is displayed in color grades from green (conserved) to red (not conserved).

The secondary structures of *Urbanus bernikerni* complex variants show the conserved *ITS2* structure usually found in eukaryotes, including four helices (labeled I-IV, Figure 7) with helix III being the longest, containing a 5′ end GGU motif, as well as the U-U mismatch associated with the *ITS2* helix II in most eukaryotes [44]. The CBC analysis identified 10–14 compensatory base pair changes among the three *Urbanus bernikerni* complex lineages in helices II and III (Table 6). Intraspecific and intragenomic CBCs were found in *Urbanus ehakernae.* CBCs were found in helix III between sequence type 06-SRNP-46891_a and four other variants: 06-SRNP-46887_c, 05-SRNP-41753, 05-SRNP-42394, 06-SRNP-46891_d (Table 6). This variant (06-SRNP-46891_a), also clusters within the *Urbanus segnestami* clade in the MP trees (Figures 6a, b).

### *Wolbachia* assays

A total of 16 individuals were positive for the *wsp* and MLST markers. This included 40% (4/10) of individuals of *Urbanus segnestami,* 100% (12/12) of the individuals of *Urbanus ehakernae*, and 0% (0/13) of *Urbanus bernikerni.* We sequenced all PCR bands for *wsp* and MLST markers. Sequences generated by extending the reverse primers for *gatB*, *coxA*, *ftsZ*, *fbpA*, and *wsp* showed heterogeneous sequence chromatograms for both *Urbanus segnestami* and *Urbanus ehakernae* specimens. The *hcpA* marker showed heterogeneous sequence chromatograms for both forward and reverse primer sequences. In all cases, the homogeneous sequence read or the highest peak was used as the consensus sequence to search the *wsp* and MLST database. All six loci matched known allelic profiles with 98-100% identity, and all infections are therefore *Wolbachia* strain 108 of Supergroup B (Table 7). Strain 108 was previously found in a butterfly, *Brangas felderi* (Goodson, 1945) (Lycaenidae), in Morona-Santiago, Ecuador [45,46]; genus *Brangas*, but not this species, does occur in ACG, though not in ecological proximity to the *Urbanus viterboana* complex.

## Discussion

This study provides genetic evidence to support the separation of what was previously called “*Urbanus belli”,* found in ACG lowland rain forest and dry forest, into a morphologically cryptic complex of three species. We found congruent patterns across 4 loci in support of three genetically distinct lineages. The fact that our cryptic complex occurs in sympatry provides indirect evidence that each lineage, when given the opportunity, does not appear to exchange genetic material. Although the intragenomic variability of *ITS2* of two individuals of *Urbanus ehakernae* could be the result of hybridization or the retention of shared ancestral polymorphisms, the ancillary ecological data collected in this study supports evolutionary distinction. *Urbanus ehakernae* is the only species of the complex restricted to rainforest habitat and this lineage appears to maintain its evolutionary trajectory regardless of gene exchange.

This study successfully implements a genetic framework for cryptic species detection and characterization. The remaining sections of this discussion focus on the application and limitations of such a framework.

### *ITS2* as a species level marker

*ITS2* is a segment of the nuclear ribosomal DNA (rDNA) cistron and has been used extensively for DNA barcoding analyses, especially in plants and fungi [47,48]. The number, size, and location of rDNA arrays can vary within species and individuals [49] and, as a result, influence the utility of the marker for phylogenetic interpretation at the species level. The process of concerted evolution homogenizes rDNA arrays and theoretically removes variability among repeats within the genome. However, intragenomic variants appear to be common [15,50,51]. For example, intragenomic variation of *ITS2* was characterized from 178 plant species [15]. The study showed an average of 35 variants per species and detected cases of shared variants in congeneric species [15]. Despite the presence of shared variants, the overall intragenomic variability was negligible, as differences were markedly smaller than those of intraspecific and interspecific differences, yielding a 97% success rate for species level identification [15]. The findings of our study agree with that of [15]; we found few shared variants between species. Although we provide inconclusive evidence as to the nature of these variants, their existence can shed light on the evolutionary history of the species complex. Similarly, studies utilizing internal transcribed spacer region 1 (*ITS1*) to confirm *COI* lineages of provisional cryptic species of parasitoid flies (Diptera, Tachinidae) and Malagasy ants [4,52,53] have also provided inconclusive evidence to address the cause of *ITS2* heterogeneity. Future work that includes closely related congeneric species over a broader geographical distribution would help to elucidate these findings.

Previous studies have utilized cloning experiments and 454-pyrosequencing to separate heterogeneous *ITS2* sequences [15,54]; the latter is analogous to a massive-scale cloning experiment. The benefits of using 454-pyrosequencing are the massive number of sequences acquired and the ability to target major and minor variants of *ITS2*[15]. More than 5000 sequences, 200-400 bp in length, were obtained after filtering and resulted in documenting over 300 variants of *ITS2*. However, a major drawback to the 454-Roche platform is the susceptibility of over/under and delayed base calling, either of which can lead to sequencing inaccuracies [55]. These inaccuracies are difficult to separate from the tendency of *ITS2*—as a non-coding region—to accumulate indels. We used a rigorous filtering process to help eliminate 454-pyrosequencing errors. Additionally, we only included variants with two or more identical sequences in the analyses, which should ensure exclusion of random sequence errors. In addition, sequencing success was low from the reverse primer direction. We suspect this is due to the preferential sequencing of primer dimers or to unperceived secondary structure in *ITS2* sequences. This limited the interpretation of ~250 bp sequences in the forward direction for intragenomic variability, and reduced the number of full-length *ITS2* variants that could be used in the MP and CBC analysis. Despite these limitations, the nMDS results suggest that the ~250 bp sequences adequately illustrate the level of genetic variability of *ITS2* reflected in the MP analysis. The quality of *ITS2* sequences recovered from NGS platforms will undoubtedly improve as NGS technology advances, ultimately increasing the utility of multi-copy genes, like *ITS2,* for phylogenetic analysis.

### Compensatory base changes in *ITS2* secondary structure

CBCs are crucial to preserve the pairings required for helices that make up the core of the *ITS2* secondary structure [56]. CBCs most likely have little causal relationship to speciation, but they may indicate that adequate evolutionary time has elapsed for speciation to have occurred [16,57]. The numbers of CBCs in *ITS2* secondary structure helices have been shown to correlate with closely related taxa being reproductively incompatible and have identified biological species of plants 93.11% of the time [15-17,56]. Previous findings that investigated the effects of *ITS2* intragenomic variability on the efficacy of CBCs to identify species found that 97% of 178 plant species could be identified, when including the relative abundance of variants [17]. The authors do state that identical *ITS2* variants were found across intra- and intergeneric species, and in a related study, 17% of the genera had congeneric species that shared *ITS2* variants [15,17]. Likewise, in our study, 39 interspecific CBCs were found between members of the *Urbanus bernikerni* complex, providing further evidence to validate this new classification (Table 6). Nevertheless, 2 intragenomic and 2 intraspecific CBCs were found within and between 2 individuals of *Urbanus ehakernae,* and other members of the lineage*.* The *ITS2* profiles of these two individuals are heterogeneous, including variants characteristic of both *Urbanus ehakernae* and *Urbanus segnestami*. As such, it is not surprising to find intragenomic and intraspecific CBCs within *Urbanus ehakernae*. As discussed in the previous section, future work focused on elucidating the nature of intragenomic and intraspecific *ITS2* variants can shed light on the evolutionary history of this cryptic complex.

### *Wolbachia* endosymbiosis

*Urbanus segnestami* and *Urbanus ehakernae* were found to share the same *Wolbachia* strain, while infection was completely absent in *Urbanus bernikerni.* This sharing between *Urbanus segnestami* and *Urbanus ehakernae* can be the result of two phenomena. The ancestor to *Urbanus segnestami* and *Urbanus ehakernae* could have been infected with this strain of *Wolbachia* and today we are observing a secondary loss of the infection in *Urbanus segnestami*, and fixation of the infection in *Urbanus ehakernae.* On the other hand, this sharing can represent unidirectional introgression of *Wolbachia* from *Urbanus ehakernae* to *Urbanus segnestami* and signify interbreeding of the lineages upon secondary contact in ACG (horizontal gene transfer).

The presence of *Wolbachia* in *Urbanus segnestami* and *Urbanus ehakernae*, and absence of infection in *Urbanus bernikerni*, can suggest that unidirectional cytoplasmic incompatibility (uniCI) [19] is a mechanism that is reducing gene flow between these pairs. However, the absence of *Wolbachia* in our investigation can also be the result of using somatic tissue, instead of reproductive tissue, as the primary source of DNA. Use of somatic tissue may decrease detection of *Wolbachia.* As a result, *Urbanus bernikerni* could be infected, but the somatic tissue tested could incorrectly test as “absent” for all 13 specimens examined, though we think that this is unlikely. To date, somatic infections have been reported in species including some in the genus *Drosophila*[58] and have been proposed to increase the likelihood of horizontal transmission routes [59]. There have been reported cases of infection being isolated to reproductive tissues in *Glossina morsitans* (Diptera, Glossinidae) females [58]. To further investigate the potential role of *Wolbachia* and speciation within the *Urbanus bernikerni* complex, reproductive, as well as somatic tissues need to be analyzed.

### Habitat distribution of the *Urbanus bernikerni* complex in the ACG

Now that we recognize three species in *Urbanus bernikerni* complex, it begins to be possible to garner some ecological sense from their intertwined and overlapping distribution within ACG. ACG is divided roughly south–north by the cloud forest-topped Cordillera Guanacaste, with Pacific coastal dry forest on the western side, Caribbean rain forest on the eastern side, and a variety of intergrades between these three major parapatric ecosystems (Figure 2). *Urbanus viterboana* is restricted to the upper cooler slopes of the volcanos, and is parapatric with the three *Urbanus bernikerni* complex species restricted to the lowlands from about 600–800 m down to sea level. *Urbanus ehakernae* is clearly a rain forest denizen and seems to have remained there (Figure 2). While *Urbanus segnestami* (Figure 2) is unambiguously able to tolerate the climatic conditions of Pacific dry forest, its omnipresence throughout ACG middle to low elevations may be a recent artifact of logging, clearing, cultivating, and pasturing of the rain forest side of ACG, an anthropomorphic force that severely insolates, heats, and dries the previously much shadier, cooler, and wetter Caribbean ground-level rain forest side of ACG. These open agroscape habitats are rich in herbaceous ruderal Asteraceae, plants fed on by the caterpillars of all three members of the *Urbanus bernikerni* complex. This presumed invasion of the (largely destroyed) rain forest ecosystem at ground level by ACG dry forest species has been repeated by many other species of insects and plants. *Urbanus bernikerni* itself (Figure 2) also appears to have originally occupied the moister and cooler marginal/intergrade portions of the ACG dry forest with the rain forest nudging in from the east, or local more moist/evergreen parts of dry forest. It will be literally many centuries before the ACG forest-in-restoration once again mimics an intact old growth forest, with its very different physical characteristics from what we see today; and we can then document what would be the “true” ecological distribution of these three sibling species in ACG. That is, however, if the habitat island of wildland ACG as a whole is not thoroughly swamped by the population and community dynamics of these three species and their host Asteraceae in the ocean of agroscape occupying Central America.

## Conclusion

Making use of DNA barcode libraries to generate hypotheses for provisional boundaries of unrecognized species can allow researchers to allocate their exploratory resources more efficiently. The discovery of the *Urbanus bernikerni* complex demonstrates that using an integrative framework, led by genetic analyses, can provide adequate evidence to confirm the presence of as-yet undescribed cryptic species. Having prior knowledge of habitat distribution played a key role in identifying this complex, and such data are highly recommended for future work. We are also careful to note that results such as these render casual specimen identification yet more difficult; we do not, for example, know which, if any, of these three cryptic species is the same as what is generally known as *Urbanus belli* throughout the Neotropics*,* since the type specimen for that name has not been DNA barcoded. Thus, extending this study to include closely related congeneric species over a broader geographical distribution is of primary interest.

## Availability of supporting data

The data sets supporting the results of this article are available in the Dryad repository, doi:10.5061/dryad.pj561 and http://datadryad.org/review?wfID=29486&token=9cf99242-b5e5-4baa-bac4-223762f92cdb[60].

## Competing interests

The authors declare that they have no competing interests.

## Authors’ contributions

CB, DHJ, WH, JMB and MH conceived the idea and designed experiments. CB, DHJ, WH, JMB, SS and MH performed experiments. CB, DHJ, WH, JMB, JFG, SS and MH analyzed and interpreted data. All authors wrote and edited the manuscript. All authors read and approved the final manuscript.
